# Single Entity Behavior of CdSe Quantum Dot Aggregates During Photoelectrochemical Detection

**DOI:** 10.3389/fchem.2021.733642

**Published:** 2021-09-10

**Authors:** Pradeep Subedi, Suman Parajuli, Mario A. Alpuche-Aviles

**Affiliations:** Department of Chemistry, University of Nevada, Reno, NV, United States

**Keywords:** CdSe quantum dot, CdSe/ZnS quantum dot, photoelectrochemistry (PEC), photooxidation, colloidal CdSe, agglomerate

## Abstract

We demonstrate that colloidal quantum dots of CdSe and CdSe/ZnS are detected during the photooxidation of MeOH, under broad spectrum illumination (250 mW/cm^2^). The stepwise photocurrent vs. time response corresponds to single entities adsorbing to the Pt electrode surface irreversibly. The adsorption/desorption of the QDs and the nature of the single entities is discussed. In suspensions, the QDs behave differently depending on the solvent used to suspend the materials. For MeOH, CdSe is not as stable as CdSe/ZnS under constant illumination. The photocurrent expected for single QDs is discussed. The value of the observed photocurrents, > 1 pA is due to the formation of agglomerates consistent with the collision frequency and suspension stability. The observed frequency of collisions for the stepwise photocurrents is smaller than the diffusion-limited cases expected for single QDs colliding with the electrode surface. Dynamic light scattering and scanning electron microscopy studies support the detection of aggregates. The results indicate that the ZnS layer on the CdSe/ZnS material facilitates the detection of single entities by increasing the stability of the nanomaterial. The rate of hole transfer from the QD aggregates to MeOH outcompetes the dissolution of the CdSe core under certain conditions of electron injection to the Pt electrode and in colloidal suspensions of CdSe/ZnS.

## Introduction

It is fundamentally interesting to understand the electrochemistry of semiconducting materials. The materials’ properties and the correlation with their reactivity have implications in energy conversion using electrochemical reactions. Since the initial reports of single NP electrochemistry, collision or nanoimpact experiments have provided information about the intrinsic kinetic parameters of electrocatalytic materials that mass transport effects may mask. Conversely, photoelectrochemistry experiments at the single entity level lag behind the analogous electrocatalytic studies. Early experiments of colloidal metal oxides include manipulating the conditions during metal electrodeposition to prepare composite materials by incorporating the metal oxide into the metal electrodeposit. Large electrodes were used to detect the photocurrent from suspended particles, or “slurries” (Dunn et al., 1981a; Dunn et al., 1981b). Our group detected TiO_2_ nanoparticles (NPs) using photocurrent in MeOH (Fernando et al., 2013). Anatase NPs collided with a Pt ultramicroelectrode (UME) which yielded stepwise current changes characteristic of single entities. In that report (Fernando et al., 2013), the observed currents were due to the photooxidation of MeOH. Fernando et al. studied dye-sensitized TiO_2_ NPs and their agglomerates in MeOH (Fernando et al., 2016) with a F-doped SnO_2_ UME. The dye was based on *cis*-bis(isothiocyanato)bis(2,2′-bipyridyl-4,4′-dicarboxylato)ruthenium(II), known as N179. Barakoti et al. studied the N719 dye/TiO_2_ system on a Pt UMEs (Barakoti et al., 2016) and two distinct responses were observed in the dark and under illumination. In the dark, at sufficiently negative potentials, dye on the TiO_2_ surface oxidizes and further oxidizes the redox-active solvent (CH_3_OH). When illuminated, the dye photooxidizes the CH_3_OH and injects electrons into the TiO_2_ NPs that the Pt UME ultimately records. Peng et al. and Ma et al. modeled transport across TiO_2_ nanostructured film that covered a metallic UME. Pent et al. detected TiO_2_ entities colliding onto a UME modified with a NP film (Peng et al., 2018b). Ma et al. (2018) used a Au/TiO_2_ UME to detect ZnO/N719 entities photooxidizing water; in these last two papers, where the authors studied the dynamics of carrier transport. Mirkin et al. (Wang et al., 2020) detected photooxidation currents from co-catalysts modified TiO_2_ NPs during water oxidation. We point out that there are electrochemical kinetic studies of semiconductor materials. Velický et al. (2016) have studied the kinetics of MoS_2_ towards the outer-sphere Ru(NH3)_6_^3+/2+^ redox couple, down to a single monolayer of SC material. Sambur et al. (2016) have mapped the spatial distribution of electron transfer on nanorods during water splitting. The authors obtained kinetic rate constants from super resolution imaging experiments. Our group is interested in studying the rate of hole transfer rate across the nanomaterials/liquid interface, and here we demonstrate that it is possible to detect the current of photooxidation for individual CdSe entities. The rate of hole transfer has been studied with transient optical techniques and electrochemistry in films, as in the case of sulfide electrolytes (Chakrapani et al., 2011).

Other systems related to semiconductor materials are the Pt NPs colliding with a Si UME covered with a TiO_2_ tunneling layer (Ahn and Bard, 2015), which displayed a large current density. There have also been studies of semiconducting materials that do not rely on photoelectrochemical detection. Tschulik et al. (2013) oxidized and reduced Fe_2_O_3_ NPs, in the so-called nano impact experiments, were able to measure the size of the particles. Our group proposed sizing of ZnO NPs based on their reduction potential (Perera et al., 2015). We also studied ZnO mass transport and electron transfer during the electrolysis of the nanomaterials (Karunathilake et al., 2020). While large bandgap materials are interesting for some applications, lower bandgap materials, such as CdSe materials, are more appealing in studies of solar energy conversion, and to the best of our knowledge, this is the first report of the stochastic electrochemistry of CdSe single entities.

Previous studies of CdSe quantum dots (QDs) include studies on ensembles of films prepared with QD (Yu D. et al., 2003; Jha and Guyot-Sionnest, 2010; Puntambekar et al., 2016; Liu et al., 2017) or the electrochemiluminescence of the material in a colloidal suspension (Myung et al., 2002). More recently, Wang et al. (2021) studied the electrocatalytic rates (activity) of single MoS_2_ quantum dots on a Ag UME towards hydrogen evolution reaction. Alshalfouh et al. (2019) studied CdSe quantum dots using impacts and single-molecule spectroscopy in aqueous solutions. They concluded that the QDs are irreversibly oxidized in the aqueous media. However, they do not lose their emissive properties after a single collision with the Pt UME, and they were capable of desorbing from the electrode surface without being significantly decomposed. There are also studies of individual semiconducting NPs with spectroscopy (Chen et al., 2017; Wang et al., 2019), but they do not follow the current from individual entities. In this paper, we present the detection of entities of CdSe quantum dots in CH_3_OH under illumination. The QDs photooxidize CH_3_OH, which is a well-known hole scavenger. The photocurrent values indicate that the current is due to the agglomerates of the QDs injecting holes into the solvent. We show that the CdSe/ZnS, because it is a more stable material under these conditions, increases the probability of detection.

## Experimental

### Chemicals

All chemicals were used as received and were purchased from Sigma Aldrich unless otherwise stated. Methanol was of spectroscopic grade and used as received. Chloroform was used as received, while acetonitrile was dried by incubation in activated alumina. For electrochemical measurements, the solvents were degassed with Ar or N_2_.

### Material Preparation

**FIGURE 1:**
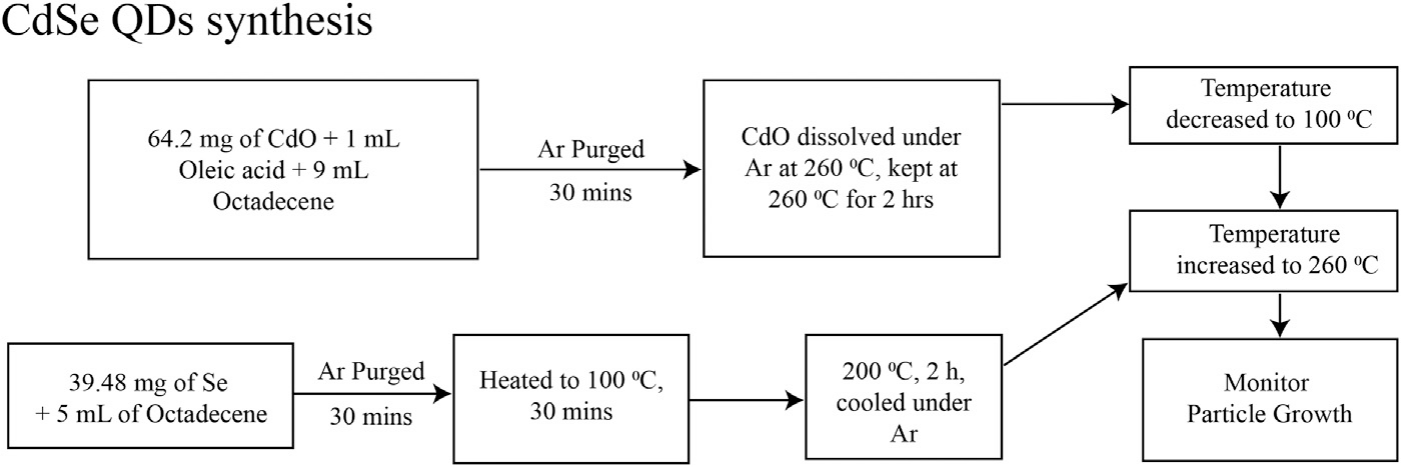
Schematics of the synthesis for CdSe QDs, modifying the procedure in ref (Jasieniak et al., 2005).

**FIGURE 2:**
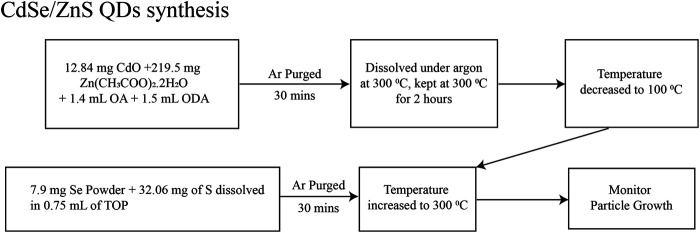
Schematics of the synthesis for CdSe QDs, modifying the procedure in ref (Bae et al., 2008).

We prepared CdSe and CdSe/ZnS QDs in colloidal solutions by modifying procedures described before. For CdSe QDs, we based our synthesis on the report in Jasieniak et al. (2005) and it is depicted in Figure 1. Briefly, the QDs were synthesized from the precursors of CdO and Se using Schlenk line techniques. The solvents and solutions were degassed and kept under a dried Ar line. Figure 2 depicts the procedure for synthesizing CdSe/ZnS, after adapting the procedure of Bae et al. (2008). This synthesis followed the usual protocols for manipulating air, and water-sensitive techniques, like the CdSe QD, described above. The precursors are CdO, Se powder, zinc acetate, and S powder.

### Material Characterization

The materials synthesized were characterized by transmission electron microscopy TEM (JOEL JEM-2100F). Photoluminescence (PL) spectra was obtained with a fluorimeter (Horiba). Dynamic light scattering (DLS) of colloidal suspensions was obtained with a NICOMP Particle Sizer 380/ZLS (PSS, Santa Barbara, CA). The electrodes’ scanning electron microscopy (SEM, Scios 2, Thermo Fisher Scientific) was performed after coating them with a Cr layer.

#### Colloidal Concentration

We estimated the colloids’ concentration from the suspension’s absorbance by calculating the value of the molar absorptivity at the first excitation peak, ε. This value was used to calculate the concentration using Beer’s law. We calculated the molar absorptivity from the optical properties, using Eqs. 1, 2 according to Yu et al. (2003b):

$$\text{ε}=5857\times D^{2.65}$$ (1)

where *D* is:

$$D=\left(1.6122\times 10^{-9}\right){\text{λ}}^{4}-\left(2.6575\times 10^{-6}\right){\text{λ}}^{3}+\left(1.6242\times 10^{-3}\right){\text{λ}}^{2}-\left(0.4277\right)\text{λ}+\left(41.57\right)$$ (2)

where λ is the wavelength of the first excitation peak.

#### Electrochemical Measurements

The setup for the electrochemical measurement has been described in detailed elsewhere (Fernando et al., 2013). Briefly, we used a three-electrode configuration with a Pt/iodide solution reference electrode. The reference electrode side of the cell included a double junction:

$${\mathrm{Pt/I}}^{-}\left(\mathrm{10mM}\right),I_{3}^{-}\left(\mathrm{10mM}\right)\mathrm{/C}H_{3}\mathrm{OH/}$$ (3)

We did not see any evidence of iodide or triiodide in the background experiments. Alternatively, we used a Ag QRE electrode. These electrodes potentials were calibrated and converted to NHE. A Xe arc lamp (Newport) illuminated a PTFE cell equipped with a silica window, and the detection was done in a commercial potentiostat (CH Instruments). We prepared the colloidal suspensions on the bench and loaded the cell; before the electrochemical experiments started, we degassed the suspensions with Ar or N_2_ for at least 20 min.

## Results and Discussion

### Material Characterization

**FIGURE 3:**
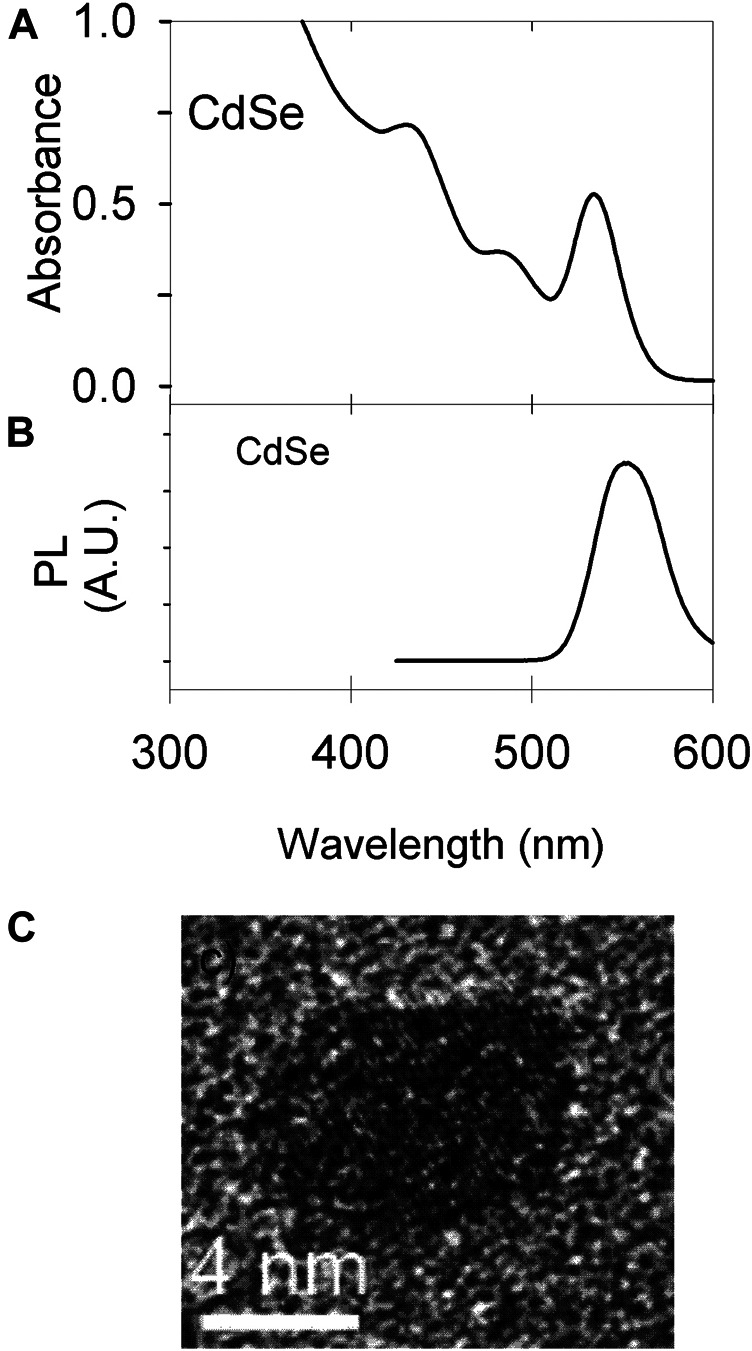
Material characterization. **(A)** UV Vis of CdSe synthesized; the colloidal concentration is 7.7 μM. **(B)** Photoluminescence of the material in a 58 μM suspension **(C)** TEM image of a single QD.

Figure 3 shows the characterization of the CdSe material by optical methods. Figure 3A shows absorption spectra and Figure 3B the photoluminescence data; both are consistent with the particle size determined by TEM of ca. 4 nm (Figure 3C).

**FIGURE 4:**
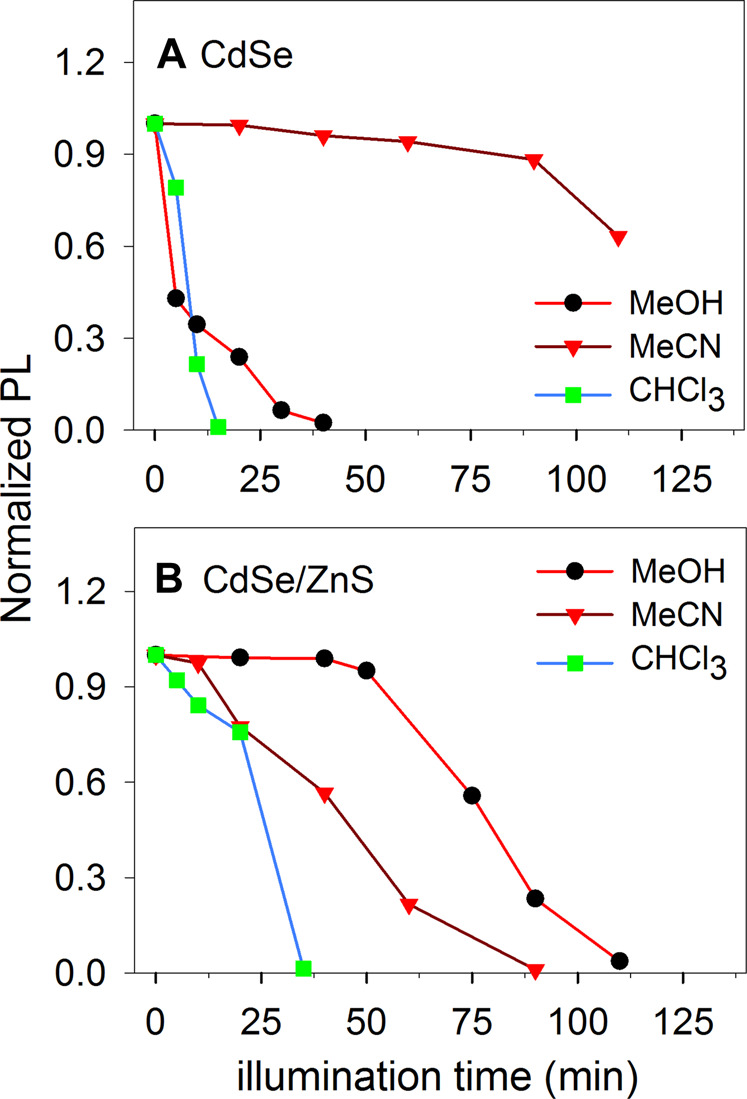
Photoluminescence of illuminated colloids of CdSe and CdSe/ZnS in different solvents with an initial concentration of ca. 10 μM.

The colloids were centrifuged and re-dispersed in methanol, acetonitrile and chloroform. Initially, we performed illumination experiments with a Xe arc lamp and monitored the materials’ fluorescence as a function of illumination time. The data in Figure 4 shows the results. Interestingly, the CdSe was stable in MeCN but not in MeOH as seen in Figure 4A, while the protected CdSe/ZnS colloids display the opposite behavior: they were stable in MeOH but not as stable in MeCN (Figure 4B). Our experiments are in nonaqueous solvents, while the stability of CdSe QDs has been studied in more detail in aqueous environments (Puzyn et al., 2009; Mulvihill et al., 2010), with some studies in toluene, e.g., (Mokari and Banin, 2003). It is interesting to note that for CdSe, the emission was more stable in CH_3_CN. In water, ligand dissociation can limit the material stability (Mulvihill et al., 2010), and could also be favorable in MeOH. We note that the electrochemical window of CH_3_CN (ca. 4.5 V), is much larger than the bandgap of the materials used in this work (ca. 2 eV), make the materials unlikely to oxidize or reduce the MeCN under illumination. For the materials, the conduction band edge would be at around −1.0 V vs. NHE (Spittel et al., 2017), while the valence band would be about +1 V vs. NHE. For MeCN, the window is typically around −2.7 V for the reduction and around +2.3 V vs. NHE for the oxidation (Bard and Faulkner, 2001). Therefore, the photogenerated electrons and holes are not expected to electrolyze the solvent.

To improve the stability of CdSe-based materials, several groups have developed methods to synthesize core-shell materials (Peng et al., 1997; Zhu et al., 2010). We studied CdSe/ZnS QDs because the layer of ZnS makes the QDs more stable and minimizes non-radiative recombination (Hines and Guyot-Sionnest, 1996). As expected, this core-shell material is more stable against photo-stimulated degeneration. However, the material eventually decays in all the solvents used, and it is more stable in MeOH, for approximately an hour or longer. In the case of chloroform, both CdSe and CdSe/ZnS were not stable in the solvent under illumination. Similar to the CH_3_CN case, for CHCl_3_ the oxidation potential is ca. +3.2 V vs NHE (Bird et al., 2020), approximately 2 V more positive than the VB edge. The reduction potential for CHCl_3_ has been reported to be ca. −1.25 vs NHE for Ag electrodes (Hoshi and Nozu, 2006), which is around 200 mV more negative than the conduction band edge for the materials. Traces of water may decrease the CdSe stability in chloroform because it is known to react with oxygen when exposed to light to produce COCl_2_, Cl_2_, and HCl, among other species (Perrin et al., 1980). Although we closed the cuvette for the experiments in Figure 4, traces of water and O_2_ may enter the colloidal suspension and produce oxidizing agents under illumination such as Cl_2_ and HCl that facilitate the oxidation of the material. In MeOH, the material is capable of oxidizing the solvent without losing its emission properties quickly.

### QD detection

**FIGURE 5:**
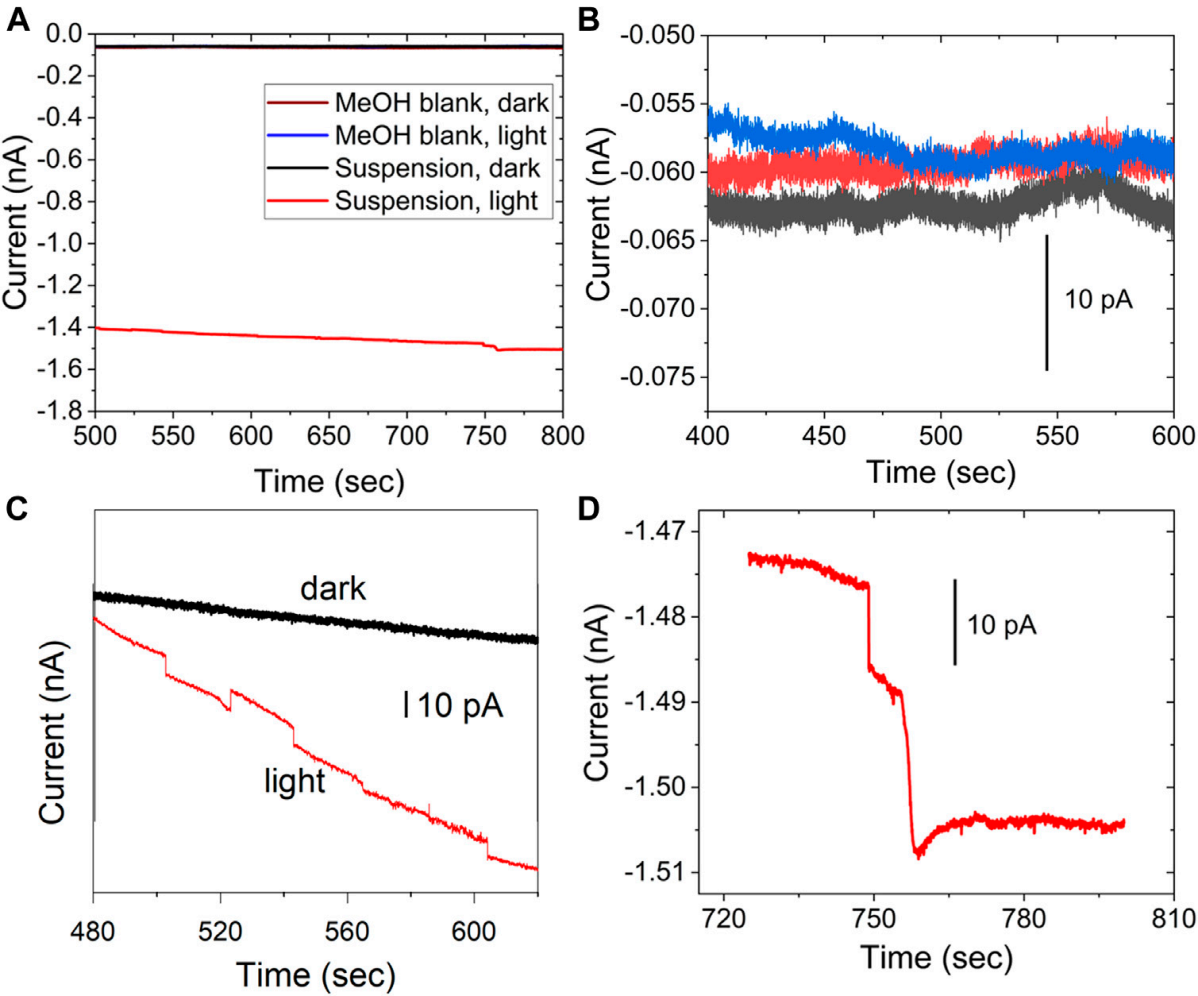
Photocurrent and control experiment to detect stochastic photocurrents for a 200 pM QD concentration in CH_3_OH, **(A)** blank MeOH in the dark (brown), blank under illumination, and control experiment for the suspension in the dark (black); all data plotted in the same scale **(B)** shows a detail for the blank and control experiments. **(C)** shows the control in the dark (black) and particle in light (red), with the data offset for clarity. **(D)** a different set of steps in detail for the data shown in a), red. 10 μm electrode, *E*_app_ = 0.2 V vs NHE.

Figure 5 shows the result for the stochastic detection of QDs suspended in MeOH and the control experiment without illumination to the colloid (*E*_app_ = 0.2 V vs NHE). Figure 5A shows the photocurrent transients observed under illumination. Note that the anodic transients, negative in the instrument’s convention, are the transients of interest. For comparison, the colloid without illumination does not show the discrete transients, consistent with the photocatalytic nature of the process, like the previous observation of anatase entities (Fernando et al., 2013). The figure also shows the methanol blank in the dark and under illumination, in the same scale as the photocurrent (red trace). The suspension in the dark and the blank are all lower in magnitude than the anodic photocurrent. Figure 5B shows the detail of the blank and controls, in a region where the currents do not show a particular trend, although due to the small current values, some regions have slopes that change during the experiment, such as the current for the colloid in the dark in Figure 5C, which has been offset to facilitate the comparison. The difference between the current under illumination and in the dark is due to photocurrent from previously deposited QDs. The material can deposit on the electrode when the UME was immersed in the suspension before the data acquisition. The staircase shape of the photocurrent in Figure 5C,D corresponds to entities photooxidizing MeOH. The stochastic electrochemistry of electrocatalytic NPs, the staircase response indicates that “sticking interactions”, are responsible (Xiao and Bard, 2007; Xiao et al., 2008). On the other hand “blips” correspond to particles that bounce off the electrode surface (Kwon et al., 2010; Kwon et al., 2011) or become inactive upon collision(Dasari et al., 2012; Dasari et al., 2014). From the data in Figure 5, entities attach irreversibly to the electrode surface while constantly turning over a product, and cathodic transients are assigned to QDs, leaving the surface or becoming inactive.

**FIGURE 6:**
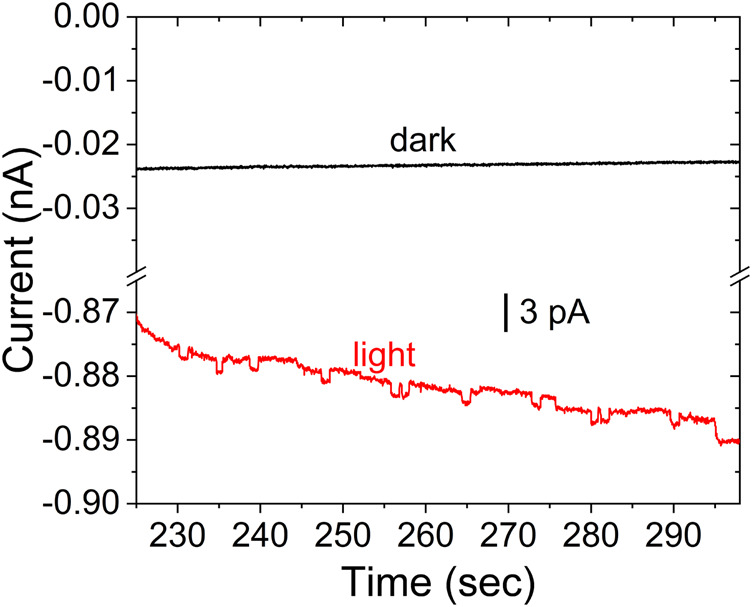
Stochastic photocurrent measurement for CdSe in MeOH under illumination (red), and control experiment for the colloid in the dark (black). All other conditions as in Figure 5.

Figure 6 shows the corresponding experimental data for CdSe without the ZnS layer for a 25 μm diameter UME. The data includes the control experiment for the suspension in the dark, which does not present any discrete current changes. As above, the difference between currents in the dark and under illumination is likely the photocurrent from CdSe already adsorbed on the electrode. In the data selected for Figure 6, many of the anodic steps that result from collisions show a return to the baseline, likely due to the lower stability of the CdSe in MeOH.

**FIGURE 7:**
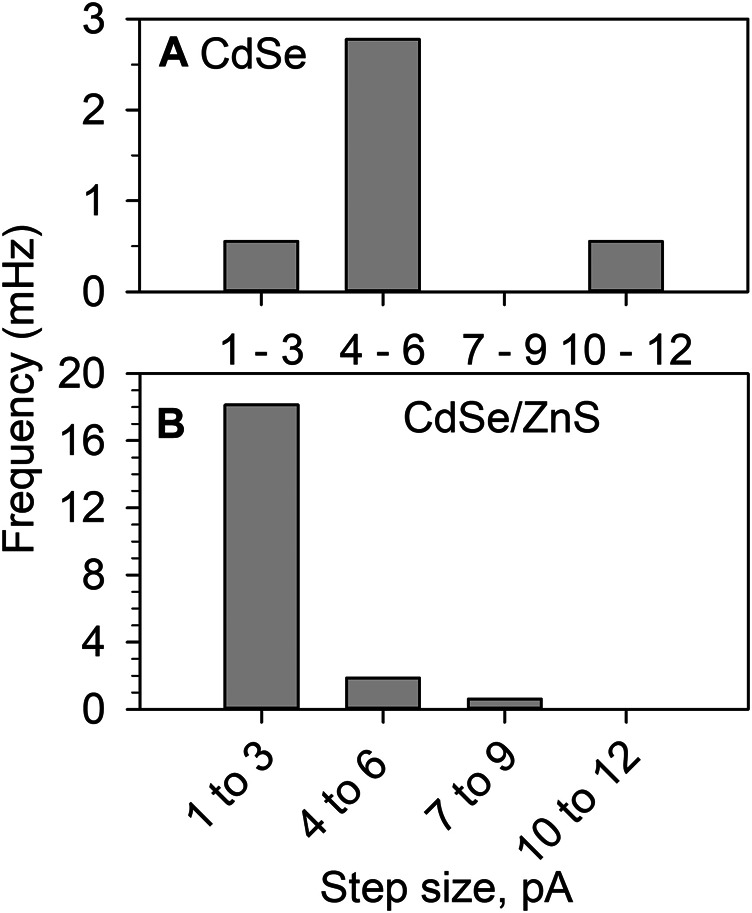
Histograms of the photocatalytic current step observed during the photooxidation of MeOH with **(A)** CdSe 1800 s of experimental time and **(B)** CdSe/ZnS for 1,600 s. All other conditions as on Figure 5.

Stochastic photoelectrochemistry yields the statistical distribution of the photocurrent. In colloidal suspensions of semiconductor NPs the diameter is expected to have a Gaussian distribution, and NPs of different sizes will have different photocurrents. Figure 7 shows the combined observed frequency of anodic steps of different sizes for the stochastic detection of both materials in MeOH. The histograms are the result of 1800 s of experimental time for CdSe and 1,600 s for CdSe/ZnS. As expected, the protected CdSe/ZnS dots (Figure 7B **)** yielded ∼5 times the frequency of the CdSe colloid (Figure 7A). This behavior is consistent with 1) the presence of more traps on the bare CdSe surface, which could cause recombination to outcompete charge separation, and 2) the CdSe being less stable in the suspensions as seen in the long-term illumination study described above (Figure 4). We used methanol in this study because it is an effective hole scavenger, and using it as a solvent facilitates QD detection (maximum MeOH concentration). The data in Figure 7 is also interesting in that for CdSe the size of the photocurrents observed is larger than for CdSe/ZnS, despite the stability issues described above. Under illumination, the product of MeOH oxidation has been reported to produce formaldehyde for TiO_2_ films (Sun and Bolton, 1996; Wang et al., 2002; Zigah et al., 2012), and under colloidal conditions, this has been recently confirmed for anatase NPs (Barakoti et al., 2021). Therefore, the photooxidation of CH_3_OH could produce HCHO through an inner sphere oxidation mechanism, which is expected to be relatively slow. For CdSe the photooxidation of MeOH is not fast enough to compete with the photo-induced dissolution of the material. If a redox mediator cannot remove holes fast enough, these can be available for the dissolution of the material (Chakrapani et al., 2011):

$$2\mathrm{CdSe}\left(h^{+}\right)\to Cd^{\mathrm{2+}}+Se^{0}$$ (4)

If the material dissolves, that will cause the removal of the oleate protecting layer. This process will cause the QDs to agglomerate, yielding a particle that will have a larger cross-section.

We note that the frequency of collision is much smaller than the expected from the diffusion-limited behavior, Eq. 5

$$f=4r_{d}D_{NP}C_{NP}^{bulk}$$ (5)

where *D*_*NP*_ is the diffusion coefficient, $C_{NP}^{bulk}$ is the bulk concentration, and *r*_d_ is the radius of UME disk. For a 5 nm QD, $C_{NP}^{bulk}$= 200 pM, and *D*_*NP*_ = 2 × 10^–6^ cm^2^ s^−1^, the frequency of collision should be > 10^5^ Hz, while the data in Figure 7 corresponds to 10^–3^ Hz. Therefore, the photocurrent is not limited by the mass transport of individual particles. This behavior is consistent with observation of our group and others (Fernando et al., 2013; Barakoti et al., 2016; Fernando et al., 2016; Peng et al., 2018a; Wang et al., 2020), although Ma et al. reported a correlation at low concentrations (Ma et al., 2018). Here, we propose that the QDs agglomerate and that the agglomerates have a much lower collision frequency.

The size of the photocurrent also points towards the detection of agglomerates or aggregates of QDs. A 5 nm diam NP should have a cross-section of ca. 2 × 10^–13^ cm^2^ to capture photons with energy larger than the bandgap; to a first approximation, we use the geometric projected area of a 5 nm QD. Our lamp’s power density is 250 mW/cm^2^, and based on the manufacturers’ data, around 16.9% of the lamp power is within the spectral region of 200–540 nm, which the QDs can absorb. We take the energy of a 250-nm photon, 8 × 10^–19^ J/photon, and assuming that this is the average energy per photon for the spectral region that the QDs can absorb. Based on the power density, there are 8.2 × 10^–15^ W that interact with the QD geometric crossection which corresponds to 10^4^ photons/QD. Suppose every interacting photon gets converted to electron-hole pairs, assuming no recombination losses, the expected photocurrent is in the order of 10^–15^ A, much smaller than the 1–10 pA in Figure 7. Therefore, aggregates are consistent with 1) photocurrents larger than expected for single QDs, 2) with the stability study and 3) with the low detection frequency.

**FIGURE 8:**
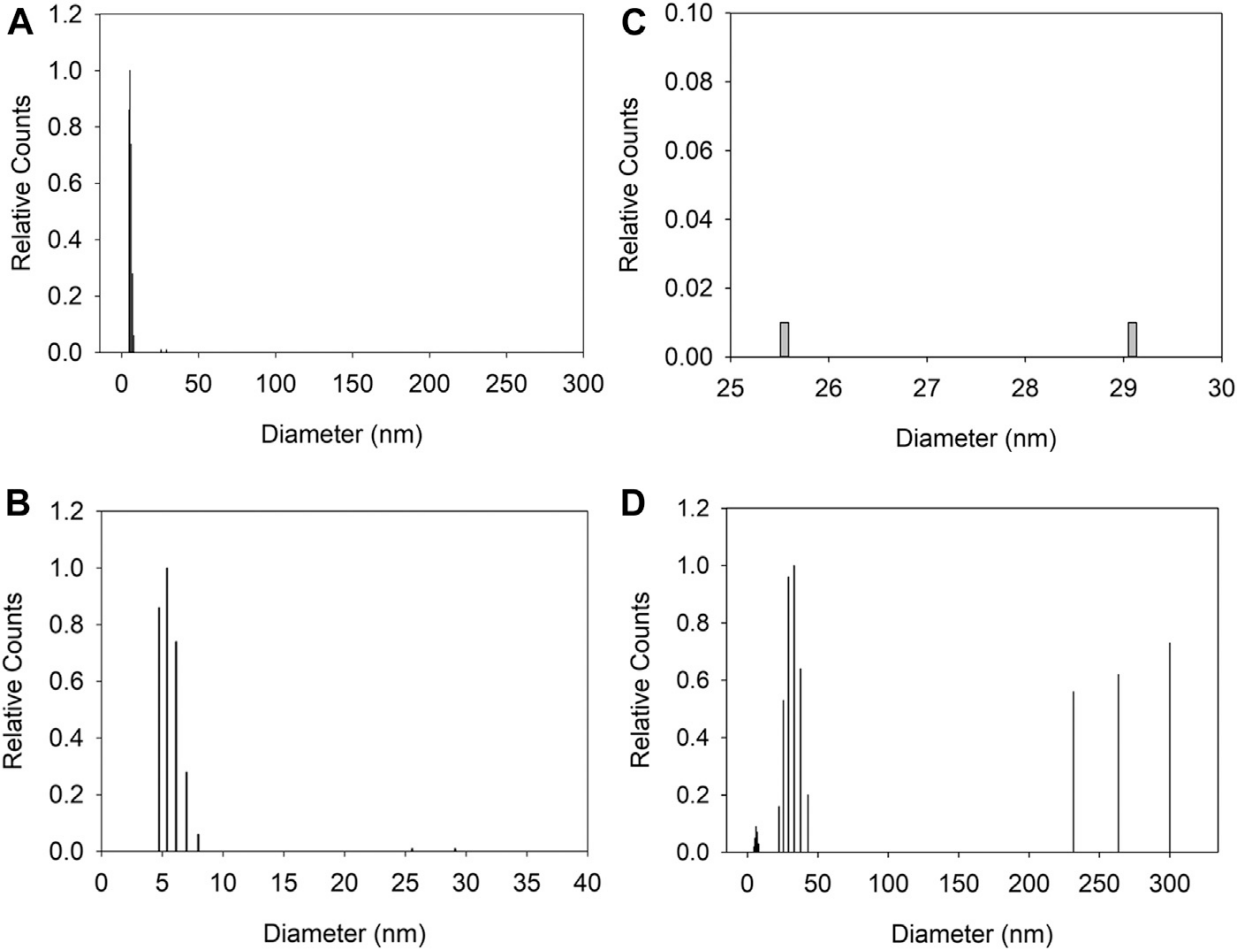
Dynamic light scattering of CdSe suspension (10 μM). **(A)** number weight of the size distribution in the 0–300 nm range. **(B,C)** show details of the number distribution in **(A)**; **(B)** is for 0–40 nm and **(C)** from 25 to 30 nm **(D)** shows the intensity weight distribution from 0 to 300 nm for the same suspension.

We performed DLS experiments on the CdSe suspensions. Figure 8 shows the size-deconvoluted results for a 10-μM CdSe suspension in CHCl_3_, before irradiation with the arc lamp. The number distribution shows that most of the concentration of NPs is distributed around the 4–10 nm size, consistent with the TEM results (Figure 8A). The details of the number distribution are shown in Figure 8B, where the NPs around the 5 nm diameter account for over 80% of the suspended NPs, and (c), where agglomerates in the 25–30 nm range are less than 0.02% of the total distribution. The intensity distribution shows much larger agglomerates that are >200 nm diam. Note that because the scattering is proportional to (Diam)^6^, these larger aggregates account for a significantly larger contribution of the scattering signal but correspond to a minuscule percentage of the total number of suspended entities.

**FIGURE 9:**
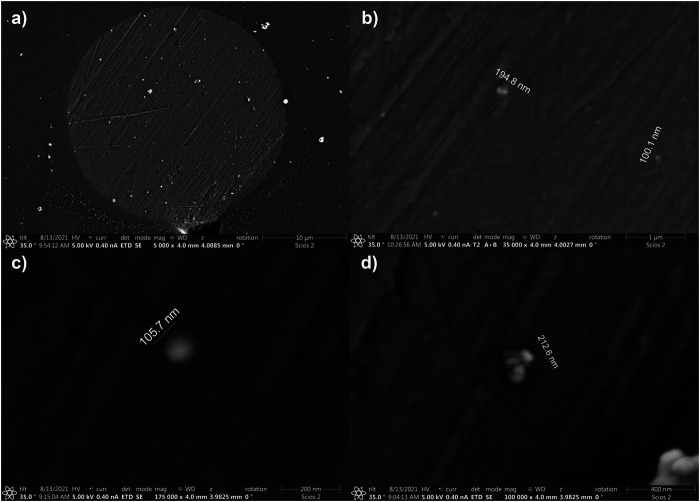
SEM of a 25 μm diam electrode after collision experiments. **(A)** a lower magnification image displaying the Pt microdisk. **(B–D)** are zooms showing agglomerates deposited at the electrode surface.

We imaged an UME after a collision experiment in a CdSe suspension, i.e., after illumination. Figure 9A shows the disk that is decorated with particles after a collision experiment. Figure 9B,C show higher magnifications of the electrode surface covered with agglomerates of QDs with sizes of 100 nm or larger. A 100-nm agglomerate, near the limit of the SEM resolution under these conditions, would correspond to entities of more than 20 QDs that have adsorbed onto the electrode surface. In summary, for the conditions of this work, we observed agglomerates before illumination by DLS, and after illumination on the electrode surface. The agglomerates are consistent with the detection of larger photocurrents.

## Conclusions

We have demonstrated photocurrent detection from single entities that form from suspended QDs during the constant irradiation of the solution. The photocurrent displays a stepwise behavior characteristic of entities adsorbing to the surface irreversibly, although some QDs leave the surface, consistent with the observations from single-molecule spectroscopy (Alshalfouh et al., 2019). In suspensions, the QDs behave differently depending on the solvent used to prepare the suspension. However, the CdSe/ZnS colloidal suspension can be stable for 1 h in MeOH, which is sufficient to detect stochastic events. The CdSe/ZnS stability indicates that the ZnS prevents carrier trapping, which allows the suspended entities to be detected. CdSe/ZnS is widely regarded as a Type I core-shell arrangement of semiconductors where the ZnS band edge energies promote electron and hole confinement within the CdSe core (Dabbousi et al., 1997). Therefore, ZnS could be a tunneling layer preventing charge transfer from the CdSe to the Pt electrode or from the material to the solution interface. However, the core-shell material is more stable in MeOH and easier to detect than the CdSe NP. The collision events display a frequency of collision that is much lower than expected based on the diffusion-limited value of dispersed QDs diffusing to the electrode surface. The photocurrent value is consistent with agglomerates due to issues of suspension stability. We are currently working on characterizing these agglomerates to deconvolute information from single NP behavior. Also, we expect to detect smaller currents with digital filtering (Gutierrez-Portocarrero et al., 2020) to enable the study of smaller agglomerates and the details of carrier trapping.

## Data Availability

The original contributions presented in the study are included in the article/Supplementary Material, further inquiries can be directed to the corresponding author.
